# Supplementary material for: Plants Play Stronger Effects on Soil Fungal than Bacterial Communities and Co-Occurrence Network Structures in a Subtropical Tree Diversity Experiment
Source: Microbiol Spectr. 2022 Apr 27;10(3):e00134-22. doi: 10.1128/spectrum.00134-22 (PMC9241759; doi:10.1128/spectrum.00134-22)
Supplement: SUPPLEMENTAL FILE 1 — Supplemental material. Download spectrum.00134-22-s001.pdf, PDF file, 1.1 MB [file spectrum.00134-22-s001.pdf]

Supplementary Materials for

Plants Play Stronger Effects on Soil Fungal Than Bacterial Communities and Co-occurrence Network Structures in a Subtropical Tree Diversity Experiment

The supplementary figures include:

FIG S1 Relative abundance of fungal and bacterial community compositions at phylum level

FIG S2 Rarefaction curves for the observed operational taxonomic units (OTUs) of soil fungi and bacteria in different tree species richness classes.

FIG S3 Non-metric multidimensional scaling (NMDS) ordination of the community composition (Aitchison dissimilarity) of fungi and bacteria.

FIG S4 Variation partitioning analysis showing the pure and shared effects of plant and abiotic factors on the community composition (Aitchison dissimilarity) of fungi and bacteria.

FIG S5 Hierarchical partitioning analysis showing the pure and shared effects of plant and abiotic factors on the community composition (Aitchison dissimilarity) of fungi and bacteria.

FIG S6 The distribution of degree for fungal and bacterial co-occurrence networks and Erdös-Rényi networks.

FIG S7 Variation partitioning analysis showing the pure and shared effects of plant and abiotic variables on the features of the co-occurrence network for fungi and bacteria.

FIG S8 Comparison of mean habitat niche breadths (*Bcom*) in bacterial and fungal communities in different tree species richness.

The additional tables include:

Table S3 Response of fungal and bacterial richness, Shannon diversity index and Simpson diversity index with plant and abiotic variables.

Table S4 Pairwise permutational multivariate analysis of variance (PerMANOVA) of fungal

and bacterial communities (Bray-Curtis dissimilarity) in different tree species richness classes.

Table S5 Hierarchical partitioning analysis of plant and abiotic factors on the community composition (Bray-Curtis dissimilarity) of fungi and bacteria.

Table S6 Hierarchical partitioning analysis of plant and abiotic factors on the community composition (Aitchison dissimilarity) of fungi and bacteria.

Table S7 Pairwise permutational multivariate analysis of variance (PerMANOVA) of fungal and bacterial communities (Aitchison dissimilarity) in different tree species richness classes.

Table S8 The number of nodes and links in the fungal and bacterial co-occurrence networks in different tree species richness classes.

Table S10 Soil variables and tree volume in different tree species richness classes.

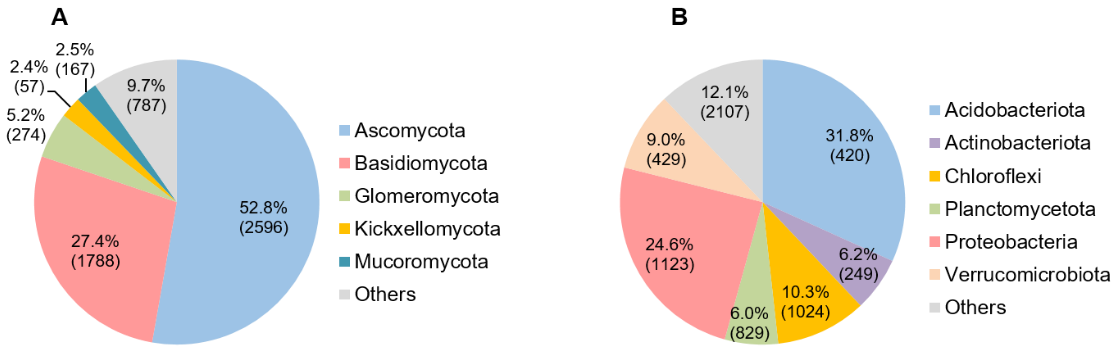

FIG S1 Relative abundance of fungal and bacterial community compositions at phylum level. **A** fungi. **B** bacteria. The fungal phyla represent < 1% of the total reads of fungi and fungi not identified to phylum level were all assigned to "Others". The bacterial phyla represent < 5% of the total reads of bacteria and bacteria not identified to phylum level were all assigned to "Others". The number of operational taxonomic units (OTUs) is shown in parentheses.

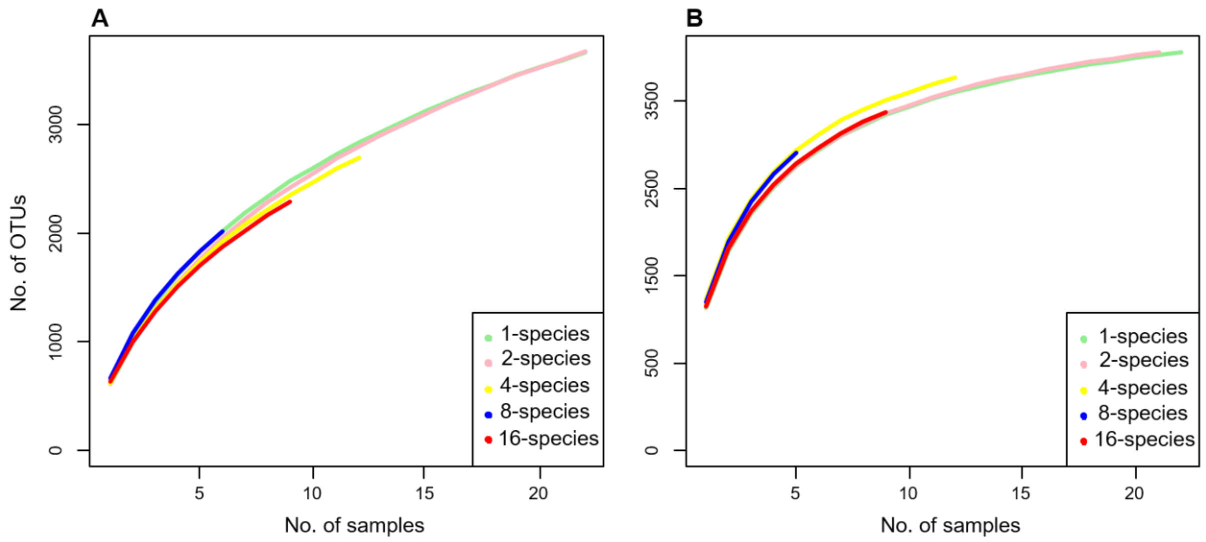

FIG S2 Rarefaction curves for the observed operational taxonomic units (OTUs) of soil fungi (**A**) and bacteria (**B**) in different tree species richness classes.

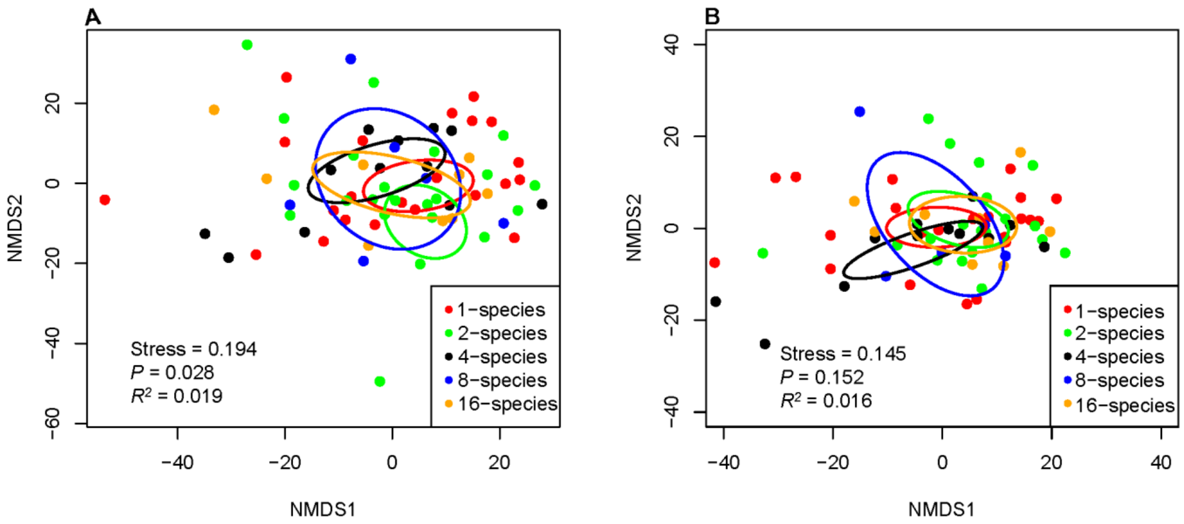

FIG S3 Non-metric multidimensional scaling (NMDS) ordination of the community composition (Aitchison dissimilarity) of fungi and bacteria. **A** fungi. **B** bacteria. Ellipses in the plots denote 95% confidence intervals for the centroids of tree species richness. Permutational multivariate analysis of variance (PerMANOVA) showed that tree species richness had a significant effect on the community composition of fungi (R^2 = 0.019, P = 0.028) but not bacteria (R^2 = 0.016, P = 0.152).

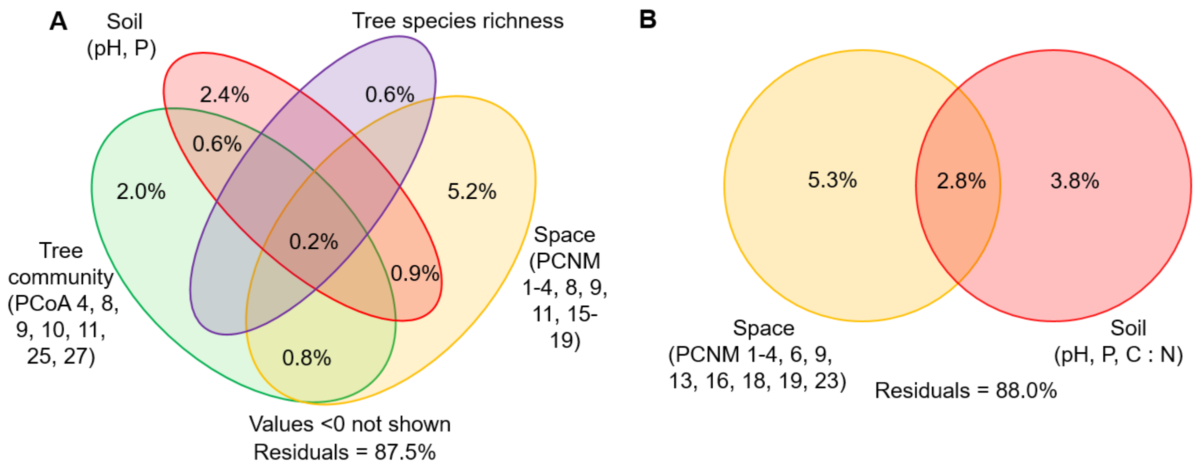

FIG S4 Variation partitioning analysis showing the pure and shared effects of plant and abiotic factors on the community composition (Aitchison dissimilarity) of fungi and bacteria. **A** fungi. **B** bacteria. Numbers indicate the proportion of explained variation. PCoA, principal coordinate analysis for the tree community; PCNM, spatial principal coordinates of neighbor matrices; P, soil total phosphorus; C, soil total carbon; N, soil total nitrogen.

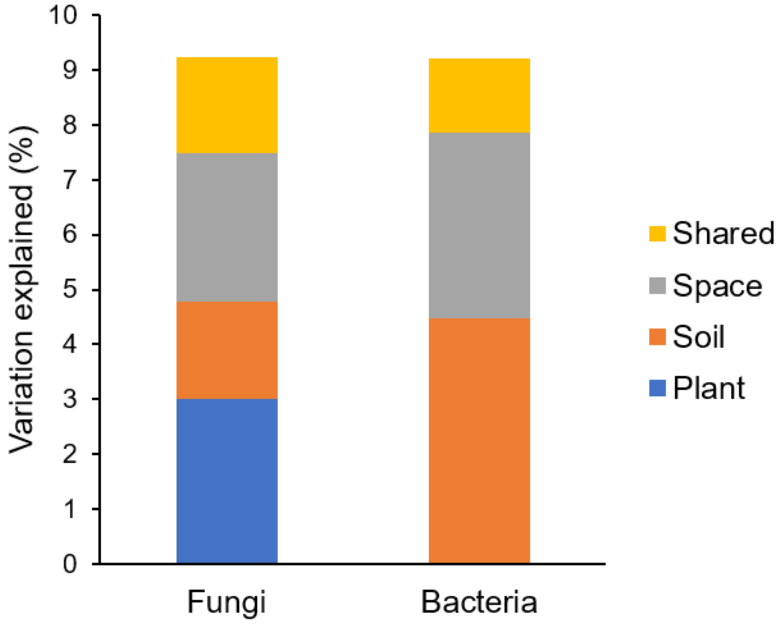

FIG S5 Hierarchical partitioning analysis showing the pure and shared effects of plant and abiotic factors on the community composition (Aitchison dissimilarity) of fungi and bacteria. Panels represent results from hierarchical partitioning analysis, aimed at identifying the percentage variance of the community composition of fungi and bacteria explained by plant (tree species richness and community composition), space and soil variables. Pure and shared variance from plant, space and soil variables in predicting the community composition of fungi and bacteria are merged in this figure for simplicity. An alternative version of this figure showing the pure and shared variance of each predictor can be found in Table S6.

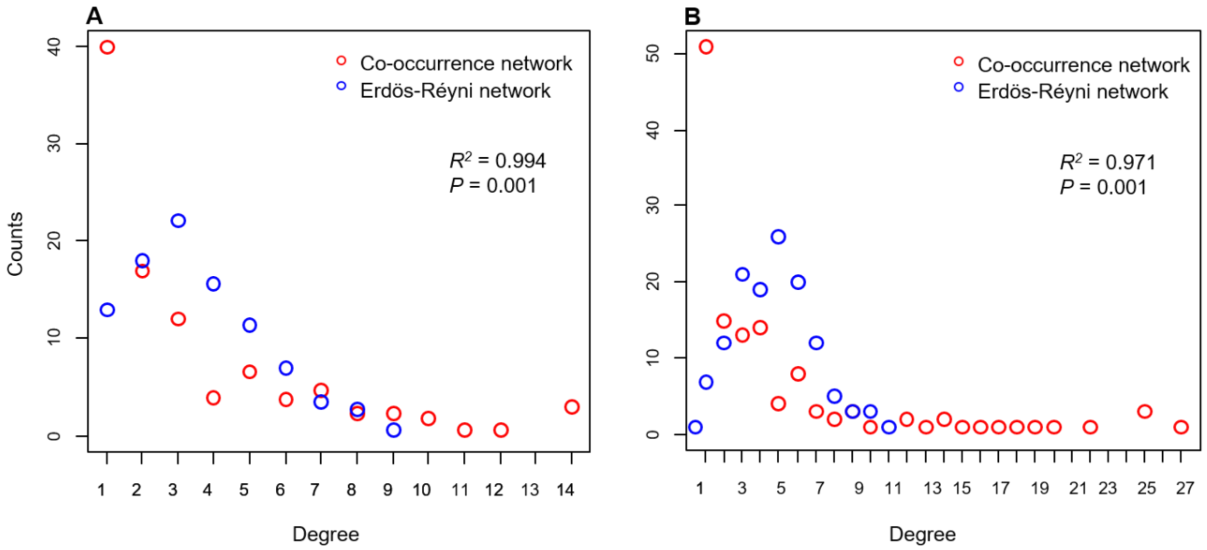

FIG S6 The distribution of degree for fungal and bacterial co-occurrence networks and Erdös-Réyni networks. **A** fungi. **B** bacteria.

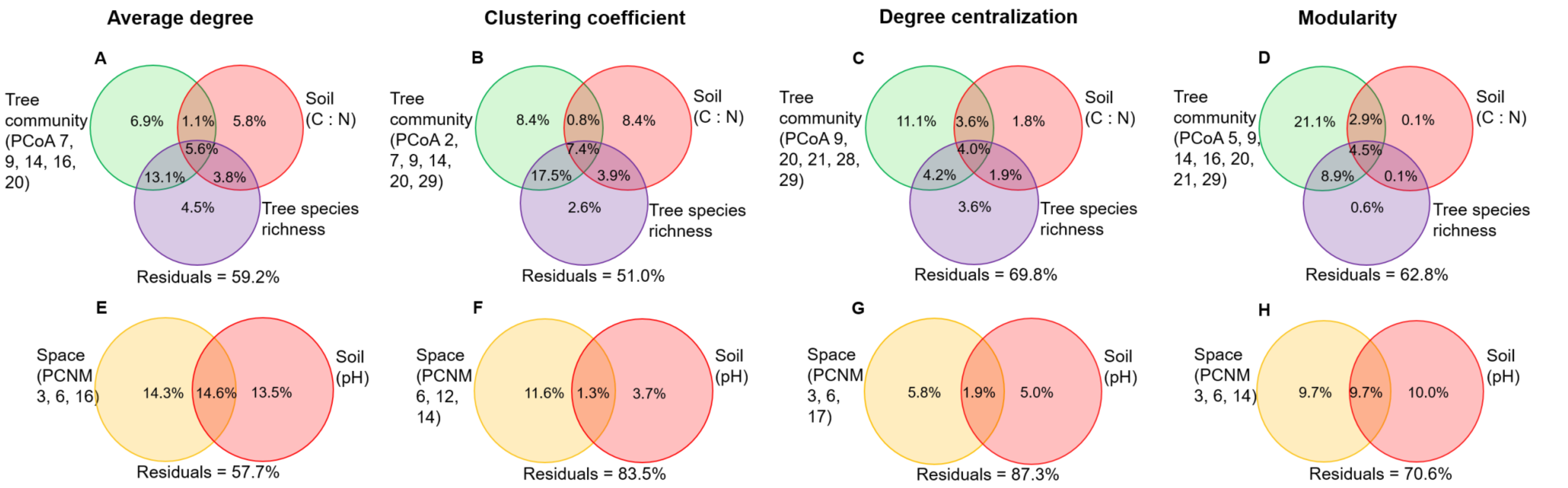

FIG S7 Variation partitioning analysis showing the pure and shared effects of plant and abiotic variables on the features of the co-occurrence network for fungi (**A-D**) and bacteria (**E-H**). Numbers indicate the proportion of explained variation. PCoA, principal coordinate analysis of tree community; PCNM, spatial principal coordinates of neighbor matrices; C, soil total carbon; N, soil total nitrogen.

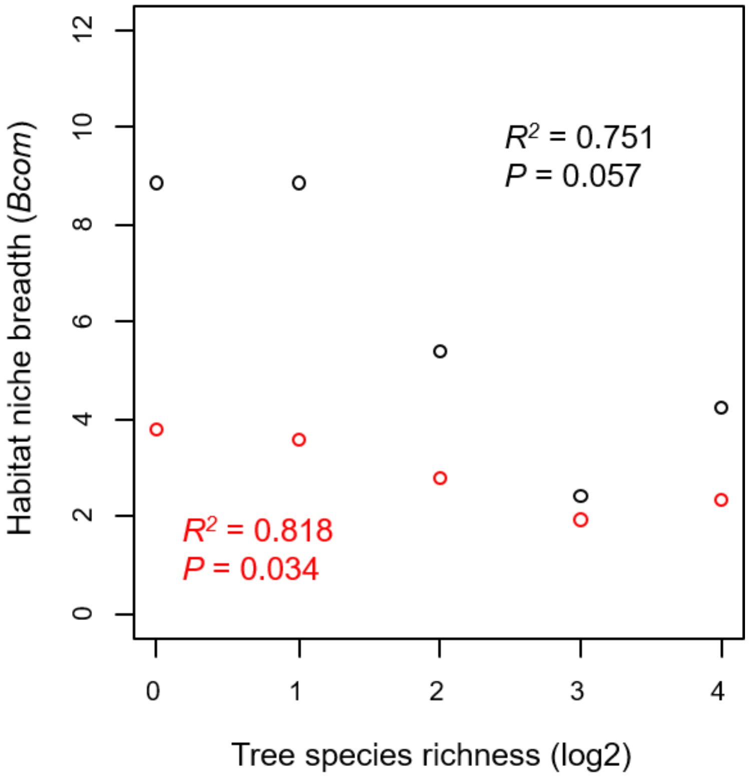

FIG S8 Comparison of mean habitat niche breadths (*Bcom*) in bacterial (black) and fungal (red) communities in different tree species richness.

Table S3 Response of fungal and bacterial richness, Shannon diversity index and Simpson diversity index with plant and abiotic variables

	Fungi						Bacteria					
	Richness		Shannon index		Simpson index		Richness		Shannon index		Simpson index	
Variables	R^2	P	R^2	P	R^2	P	R^2	P	R^2	P	R^2	P
Tree species richness	<0.001	0.938	0.008	0.432	0.006	0.476	0.001	0.834	<0.001	0.820	0.008	0.456
Tree volume	0.015	0.314	0.027	0.167	0.011	0.387	0.003	0.651	<0.001	0.574	0.002	0.701
pH	0.034	0.122	0.002	0.738	0.015	0.297	0.001	0.777	0.034	0.127	0.033	0.133
C	0.014	0.328	<0.001	0.820	0.012	0.371	<0.001	0.880	<0.001	0.914	0.007	0.489
N	0.013	0.350	<0.001	0.869	0.009	0.413	<0.001	0.538	<0.001	0.907	0.006	0.497
P	<0.001	0.892	0.054	0.051	0.047	0.068	<0.001	0.506	0.007	0.484	0.013	0.342
C : N	0.002	0.727	0.002	0.714	0.002	0.694	0.001	0.795	0.001	0.843	0.057	0.811
C : P	0.002	0.681	<0.001	0.958	0.001	0.846	<0.001	0.960	0.004	0.599	0.018	0.271
N : P	0.001	0.766	<0.001	0.947	0.002	0.723	0.002	0.975	0.004	0.596	0.017	0.289

P, soil total phosphorus; C, soil total carbon; N, soil total nitrogen

Table S4 Pairwise permutational multivariate analysis of variance (PerMANOVA) of fungal and bacterial communities (Bray-Curtis dissimilarity) in different tree species richness classes

	Fungi			Bacteria		
Tree species richness pairs	F model	R^2	*P*	F model	R^2	*P*
Monoculture vs. 2-species	0.959	0.022	0.735	1.057	0.025	0.324
Monoculture vs. 4-species	1.475	0.044	**0.020**	1.445	0.043	0.102
Monoculture vs. 8-species	1.318	0.048	**0.048**	0.901	0.034	0.553
Monoculture vs. 16-species	1.498	0.049	**0.020**	1.768	0.057	0.350
2-species vs. 4-species	1.349	0.040	**0.033**	1.187	0.036	0.194
2-species vs. 8-species	1.170	0.043	0.070	0.714	0.028	0.880
2-species vs. 16-species	1.259	0.041	**0.040**	1.005	0.034	0.366
4-species vs. 8-species	0.934	0.055	0.735	0.763	0.048	0.727
4-species vs. 16-species	0.976	0.048	0.703	1.126	0.055	0.260
8-species vs. 16-species	0.912	0.065	0.735	0.826	0.064	0.677

Table S5 Hierarchical partitioning analysis of plant and abiotic factors on the community composition (Bray-Curtis dissimilarity) of fungi and bacteria

Fungi				
Variables	Unique effect	Average shared effect	Individual effect	Percentage of individual effect towards total explained variation (%)
Tree species richness	0.0079	-0.0009	0.0070	5.60
P	0.0041	0.0145	0.0186	14.88
pH	0.0157	0.0122	0.0279	22.32
PCoA4	0.0064	-0.004	0.0060	4.80
PCoA8	0.0033	0.0014	0.0047	3.76
PCoA9	0.0126	-0.0025	0.0101	8.08
PCoA10	0.0062	0.0022	0.0084	6.72
PCoA11	0.0021	0.0008	0.0029	2.32
PCNM1	0.0071	-0.0013	0.0075	6.00
PCNM2	0.0064	0.0001	0.0065	5.20
PCNM3	0.0082	0.0023	0.0105	8.40
PCNM4	0.0060	0.0000	0.0060	4.80
PCNM7	0.0020	0.0014	0.0034	2.72
PCNM9	0.0025	0.0033	0.0058	4.64
Total explained variation			0.125	
Bacteria				
P	0.0086	0.0116	0.0202	15.66
pH	0.0453	0.0164	0.0617	47.83
C : N	0.0050	0.0021	0.0071	5.50
PCNM3	0.0078	0.0062	0.0140	10.85
PCNM6	0.0094	0.0015	0.0109	8.45
PCNM9	0.0006	0.0059	0.0065	5.04
PCNM16	0.0002	0.0027	0.0029	2.25
PCNM18	0.0045	0.0010	0.0055	4.26
Total explained variation			0.129	

PCoA, principal coordinate analysis for the tree community; PCNM, spatial principal coordinates of neighbor matrices; P, soil total phosphorus; C, soil total carbon; N, soil total nitrogen.

Table S6 Hierarchical partitioning analysis of plant and abiotic factors on the community composition (Aitchison dissimilarity) of fungi and bacteria

Fungi				
Variables	Unique effect	Average shared effect	Individual effect	Percentage of individual effect towards total explained variation (%)
Tree species richness	0.0061	-0.0004	0.0057	6.20
P	0.0091	0.0067	0.0158	17.17
pH	0.0087	0.0109	0.0196	21.30
PCoA4	0.0064	-0.0001	0.0031	3.37
PCoA8	0.0036	0.0010	0.0046	5.00
PCoA9	0.0089	-0.0022	0.0067	7.28
PCoA10	0.0046	0.0021	0.0067	7.28
PCoA11	0.0004	0.0008	0.0012	1.30
PCNM3	0.0063	0.0023	0.0086	9.35
PCNM4	0.0051	-0.0001	0.0050	5.43
PCNM8	0.0049	-0.0009	0.0040	4.35
PCNM9	0.0007	0.0023	0.0030	3.26
PCNM11	0.0045	-0.0009	0.0036	3.91
PCNM15	0.0055	-0.0007	0.0048	5.22
Total explained variation			0.092	
Bacteria				
P	0.0094	0.0074	0.0168	18.26
pH	0.0279	0.0108	0.0387	42.07
C : N	0.0074	-0.0011	0.0063	6.85
PCNM4	0.0067	0.0006	0.0073	7.93
PCNM6	0.0068	0.0008	0.0076	8.26
PCNM 10	0.0046	0.0021	0.0067	7.28
PCNM13	0.0048	0.0000	0.0048	5.22
PCNM16	0.0006	0.0011	0.0017	1.85
PCNM19	0.0062	-0.0005	0.0057	6.20
PCNM23	0.0041	-0.0005	0.0036	3.91
Total explained variation			0.092	

PCoA, principal coordinate analysis for the tree community; PCNM, spatial principal coordinates of neighbor matrices; P, soil total phosphorus; C, soil total carbon; N, soil total nitrogen.

Table S7 Pairwise permutational multivariate analysis of variance (PerMANOVA) of fungal and bacterial communities (Aitchison dissimilarity) in different tree species richness classes

	Fungi			Bacteria		
Plant richness pairs	F model	R^2	*P*	F model	R^2	*P*
Monoculture vs. 2-species	0.848	0.020	0.917	0.949	0.023	0.543
Monoculture vs. 4-species	1.082	0.033	0.242	1.132	0.034	0.179
Monoculture vs. 8-species	1.114	0.041	0.195	0.920	0.035	0.629
Monoculture vs. 16-species	1.190	0.039	0.089	1.229	0.041	0.096
2-species vs. 4-species	1.211	0.036	0.053	1.081	0.034	0.202
2-species vs. 8-species	1.081	0.040	0.217	0.831	0.033	0.928
2-species vs. 16-species	1.126	0.037	0.144	0.946	0.033	0.585
4-species vs. 8-species	0.931	0.055	0.621	0.854	0.054	0.813
4-species vs. 16-species	0.969	0.049	0.450	1.017	0.051	0.342
8-species vs. 16-species	0.825	0.060	0.906	0.894	0.069	0.719

Table S8 The number of nodes and links in the fungal and bacterial co-occurrence networks in different tree species richness classes

Tree species richness	1	2	4	8	16
**Fungi**					
Number of nodes	68	60	59	55	49
Number of links	71	67	46	46	36
**Bacteria**					
Number of nodes	104	102	102	98	102
Number of links	264	263	263	260	263

Table S10 Soil variables and tree volume in different tree species richness classes

Tree species richness	P (g kg^{-1})	C (g kg^{-1})	N (g kg^{-1})	pH	C : N	C : P	N : P	Tree volume (m^3 ha^{-1})
1	0.28 ±0.09	64.01 ±25.90	4.80 ±1.85	3.83 ±0.10	13.24 ±0.80	257.25 ±138.38	19.33 ±10.09	0.14 ±0.12
2	0.30 ±0.12	60.88 ±34.20	4.75 ±2.51	3.81 ±0.14	12.76 ±0.92	241.51 ±155.21	18.89 ±11.69	0.17 ±0.17
4	0.29 ±0.10	69.53 ±16.27	5.83 ±1.37	3.83 ±0.12	11.93 ±0.25	307.19 ±282.22	25.81 ±23.81	0.23 ±0.16
8	0.28 ±0.03	43.33 ±8.33	3.52 ±0.66	3.80 ±0.07	12.32 ±0.35	158.79 ±40.12	12.89 ±3.32	0.29 ±0.19
16	0.33 ±0.10	35.07 ±14.10	2.87 ±1.16	3.71 ±0.10	12.30 ±1.14	116.88 ±69.31	9.46 ±5.41	0.24 ±0.16

Data are average value ±standard deviation. P, soil total phosphorus; C, soil total carbon; N, soil total nitrogen
